# Impact of Edaphic and Climatic Factors on *Thymus pulegioides* Essential Oil Composition and Potential Prevalence of Chemotypes

**DOI:** 10.3390/plants11192536

**Published:** 2022-09-27

**Authors:** Vaida Vaičiulytė, Kristina Ložienė, Ričardas Taraškevičius

**Affiliations:** 1 Nature Research Centre, Institute of Botany, Žaliųjų Ežerų Str. 47, 08406 Vilnius, Lithuania; 2 Nature Research Centre, Institute of Geology and Geography, Akademijos Str. 2, 08412 Vilnius, Lithuania

**Keywords:** large thyme, essential oils, geraniol, carvacrol, thymol, α-terpinyl acetate, linalool, soil chemistry, climatic conditions

## Abstract

Intraspecific chemical polymorphism is characteristic of essential oil bearing *Thymus pulegioides* (Lamiaceae). Soil chemical composition and climatic conditions can influence not only quantitative and qualitative composition of essential oils, but also on prevalence of different chemotypes in space. The purpose of study was to determine the impact of edaphic and climatic factors on *T. pulegioides* essential oil composition and potential chemotypes prevalence. It were investigated 131 habitats of *T. pulegioides* in all ten climatic sub-districts of Lithuania. Essential oils were isolated by hydrodistillation and analysed by GC-FID and GC-MS. The content of humus was estimated by oxidation method, the content of mobile potassium (K_2_O) and mobile phosphorus (P_2_O_5_)—flame photometry using 0.2 M HCl solution, the soil pH—electrometrically and 15 elements of soil—by energy-dispersive X-ray fluorescence. The meteorological data (temperature, rainfall, photosynthetically active solar radiation and sunshine duration) were obtained from the meteorological bulletins. Results showed that humus in soil positively, manganese and cobalt—negatively influenced on the essential oil accumulation in *T. pulegioides*. Carvacrol was the most common compound in the essential oil of *T. pulegioides*; higher amounts of sulphur and mobile phosphorus, lower amounts of sodium in soil, higher rainfall in blooming period are favourable for this compound and for potential prevalence of carvacrol chemotype. Geraniol was frequent in the eastern and the central part of Lithuania, where the temperature in vegetation season is higher, and in soils characterised by higher and lower amounts of sodium and chlorine, respectively. Thymol, linalool and α-terpinyl acetate were rare. Edaphic and climatic conditions can differently influence on essential oil yield and chemical composition of species as well as on potential prevalence of different chemotypes of same species in space.

## 1. Introduction

The large thyme (*Thymus pulegioides* L., Lamiaceae) is medical, aromatic, essential oil bearing plant, distributed in all territory of Europe [1]. The main volatile compounds of *T. pulegioides* are thymol, carvacrol, geraniol, linalool, α-terpinyl acetate, fenchone, and cis-sabinene hydrate. These compounds can amount significant percentages in essential oils (as well as determinate chemotypes of *T. pulegioides*) [2,3,4], are biologically active and have a fairly wide range of application. The monoterpenic phenol thymol together with isomer carvacrol have wide spectrum of antioxidant and antimicrobial activity, therefore, are widely used in pharmaceutical and food industries [5,6,7,8,9]. The acyclic monoterpene alcohols geraniol and linalool are widely used in cosmetics, perfumery for their intense aroma and as the insect repellents [10,11,12]. The α-terpinyl acetate has antibacterial properties [13,14].

Composition of essential oils of species of genus *Thymus* is determined by genetical factors but it also can be influenced by temperature, amount of precipitation, sunshine duration, soil chemical composition, altitude above the sea level and other environmental factors [15,16,17,18,19,20,21]. Same environmental factors can unequally effect on different volatile compounds. For example, cultivation of phenolic and non-phenolic chemotypes of large thyme in some locality showed that higher temperature in April–July stimulates essential oil and geraniol accumulation, meanwhile higher photosynthetically active solar radiation inhibites geraniol accumulation in plants of geraniol chemotype, photosynthetically active solar radiation stimulates and higher temperature inhibites essential oil accumulation in plants of carvacrol chemotype, higher temperature in April–July stimulates essential oil accumulated in linalool chemotype of *T. pulegioides* [22]. Therefore, very likely that same environmental factors could impact different effect on distribution of different chemotypes in space.

The edaphic and climatic conditions in the territory of Lithuania are different. The marine climate is more pronounced in the western part, while the continental climate in the eastern part of Lithuania; therefore climatic sub-districts distinguished in Lithuania differ by the average annual temperature, the amount of precipitation per year, the duration of snow preservation [23]. Higher amounts of humus, mobile phosphorus, mobile potassium and many micro-elements are characteristic of soils in the central part of Lithuania, higher soil acidity—characteristic of the western part of Lithuania [24,25]. Therefore, these differences of climatic and edaphic factors in the territory of Lithuania can affect not only the quantitative and qualitative composition of essential oils of *T. pulegioides*, but also different prevalence of *T. pulegioides* chemotypes in space. As was mentioned above, essential oils extracted from different *T. pulegioides* chemotypes characterized by different properties and are used in different practices. Raw material of *T. pulegioides* different chemotypes can be both grown in culture and harvested in natural habitats. Therefore, knowledges of effect of edaphic and climatic conditions on prevalence of *T. pulegiodes* chemotypes could be useful both for cultivation of different chemotypes and collection of raw material of different chemotypes in the natural habitats. The purpose of study was to determine the influence of edaphic and climatic factors on *T. pulegioides* essential oil composition and chemotypes prevalence.

## 2. Results

### 2.1. Analysis of Composition of Thymus pulegioides Essential Oils and Classification of Thymus pulegioides Habitats According to Main Chemical Compounds of Essential Oils

**Figure 1:**
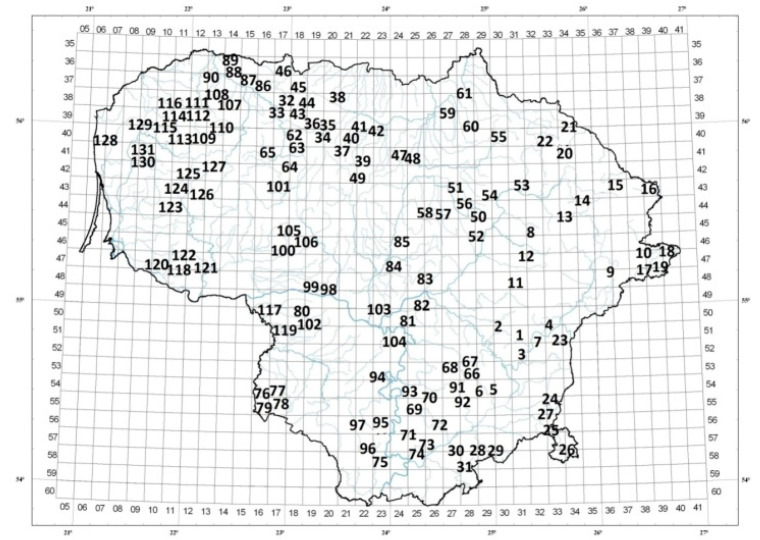
Distribution of investigated *Thymus pulegioides* habitats in Lithuania. Numbers of habitats of climatic sub-district: Aukštaitija: 1–4, 7–23; Dzūkija: 5, 6, 24–31, 66–75; Mūša-Nevėžis: 32–65, 83–85, 101, 105, 106; the Nemunas Lowland: 80–82, 91–100, 102–104, 117–122; Sūduva: 76–79; Venta: 86–90; Žemaičiai: 107–116, 123–127, 129, 130; Pajūris: 128; Pajūris Lowland: 131.

Quantitative analysis of essential oils of *T. pulegioides* raw materials, collected from 131 habitats in all territory of Lithuania, showed that mean amount of essential oil was 0.61 ± 0.21% and varied from 0.20% in habitat no. 45 (Kruopiai, Mūša-Nevėžis sub-climatic district) to 1.32% in habitat no. 121 (Bardėnai, the Nemunas Lowland climatic sub-district) (Figure 1).

In the essential oils of *T. pulegioides* has been identified 55 chemical compounds. Nineteen chemical compounds exceeded 4% in the essential oils of *T. pulegioides*. Carvacrol was abundant and the most frequent chemical compound (17.66 ± 9.43%) in the essential oils of *T. pulegioides*. This chemical compound was detected in plant material collected from all investigated habitats, except habitat no. 64 (Padubysys, Mūša-Nevėžis climatic sub-district). The variation of this phenol was the lowest (CV = 53%) in comparison with other analysed chemical compounds of essential oils. The percentage of thymol (carvacrol isomer) was 5.5 times lower compared to carvacrol, though this compound was established even in ¾ of investigated habitats. The mean percentage of γ-terpinene (precursor of thymol and carvacrol) was similar to percentage of carvacrol, meanwhile the mean percentage of other precursor p-cymene was lower compared with γ-terpinene. The mean percentage of geraniol in essential oils of *T. pulegioides* was 2.7 times lower than mean percentage of carvacrol (Table 1). Linalool was established in most habitats, but amounted about 1% of essential oils often. α-Terpinyl acetate was established only in 1/3 of investigated habitats. The mean percentages of linalool and α-terpinyl acetate amounted less than 2% of essential oil but the variation of amounts of these compounds was the highest compared with other chemical compounds (Table 1). However, the max percentage of linalool and α-terpinyl acetate was 1.2–1.4 time higher than the max percentage of carvacrol and geraniol. 

**Figure 2:**
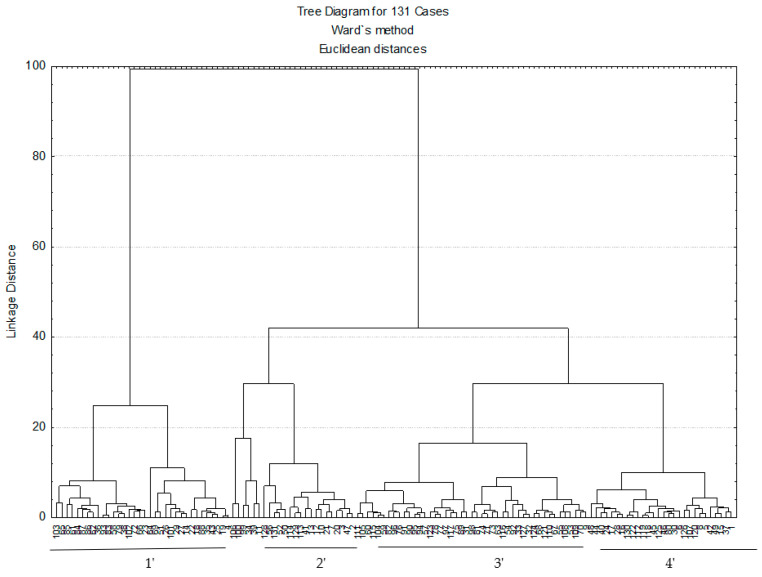
Two dimensional dendrogram of *Thymus pulegioides* habitats by chemotypes determining the main chemical compounds of essential oils.

**Figure 3:**
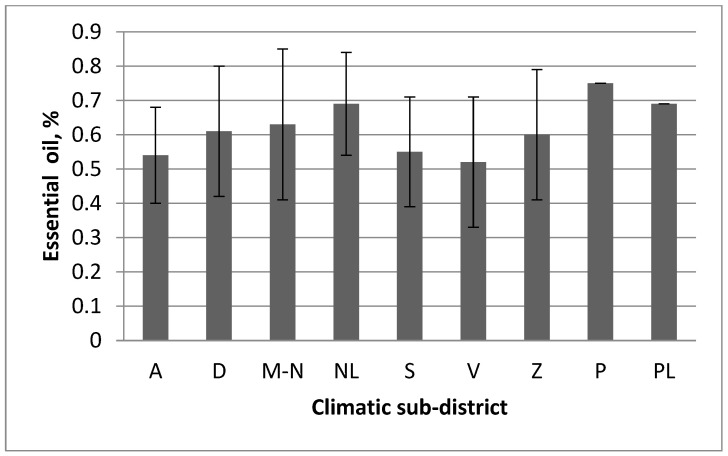
Comparison of amount of essential oil in *Thymus pulegioides* habitats in different Lithuania climatic sub-districts. Explication: A—Aukštaitija climatic sub-district, D—Dzūkija, M-N—Mūša Nevėžis, NL—the Nemunas Lowland, S—Sūduva, V—Venta, Z—Žemaičiai, P—Pajūris, PL—Pajūris Lowland. The statistical significances between climatic sub-districts has not been established.

Four clusters of *T. pulegioides* habitats were distinguished by cluster analysis according to percentage of above-mentioned main compounds of chemotypes of *T. pulegioides* and their precursors (Figure 2). Cluster 1′ (34 habitats) distinguished habitats with the highest percentages of geraniol and biogenetically related compounds (geranial, nerol and neral). Geraniol in the habitats of this cluster was 6–8 times, geranial—4–10 times, nerol—4–13 times and neral—4–15 times higher than in the habitats of other three clusters (Table 2, Figure 2). There variation of geraniol, geranial, nerol and neral percentages between habitats was less in cluster 1′ than in other clusters (Table 2). Tukey’s post-hoc demonstrated that cluster 1′ significantly (*p* < 0.05) differed from other clusters by the amounts of geraniol, geranial, nerol and neral. The highest geraniol percentage was found in habitat no. 69 (Kruonis) belonging to Dzūkija climatic sub-district (Figure 1 and Figure 2. Thymol dominated in the essential oils of *T. pulegioides* samples collected in habitats of cluster 2′. Amounts of thymol in essential oils of *T. pulegioides* were 4–7 times higher in habitats of this cluster in comparison with other clusters. The variation of thymol and thymol methyl ether between habitats of this cluster was the lowest (Table 2). The highest amount of thymol was in habitat no. 128 (Palanga, Pajūris climatic sub-district) (Figure 1 and Figure 3). Tukey’s post-hoc showed significant differences between cluster 2′ and other clusters according to percentage of thymol and thymol methyl ether (*p* < 0.05). In cluster 2′ merged only 17 habitats. Clusters 3′ and 4′ characterised by high amount of carvacrol. Tukey’s post-hoc showed that habitats of cluster 3′ significantly (*p* < 0.05) differed from other clusters by the amount of carvacrol and γ-terpinene in the essential oil of *T. pulegioides*. The highest amount of carvacrol was established in habitat no. 31 (Puvočiai, Dzūkija climatic sub-district), where it reached 48.00% in essential oil (Figure 1 and Figure 2). Meanwhile the amount of carvacrol was about 10% lower in cluster 4′ than in cluster 3′, there percentage of precursor p-cymene was 2 times higher than in cluster 3′. The highest amount of p-cymene was established in habitat no. 48 (Liberiškės, Mūša–Nevėžis climatic sub-district) of cluster 4′ (Figure 1 and Figure 2). Tukey’s post-hoc showed that cluster 4′ significantly (*p* < 0.05) different from cluster 1′ by the amount of p-cymene. Habitats no. 11, no. 34, no. 39, no. 99, no. 100 and no. 106 were not assigned for any cluster and were analysed separately (Figure 2). α-Terpinyl acetate was dominated in habitats no. 34, no. 39 and no. 99, linalool—in habitats no. 106 and no. 100. 

### 2.2. Soil Chemistry and Its Effect on Composition of Thymus pulegioides Essential Oils 

The soil pH varied from slightly acid (pH = 5.1) to medium alkaline (pH = 8.3) in investigated *T. pulegioides* habitats, however neutral or slightly alkaline values were the most common. The mean values of soil pH were similar in all four clusters (Table 3). The mean amount of humus in soils of clusters was 2.5–2.8%, the mean amount of mobile potassium and phosphorus—100–135 mg/kg (Table 3); such values of mobile phosphorus and potassium are considered as medium [25]. Silicon was characterised by the largest amount and the lowest variation of amount in the soils of clusters of *T. pulegioides* habitats in comparison with other investigated 14 chemical elements of soil. Amounts of cobalt and copper were the lowest in soils of investigated habitats (Table 4). 

The results showed that the quantitative composition of essential oils of *T. pulegioides* investigated samples (N = 131) positively correlated with the amount of humus and negatively with amounts of cobalt and manganese in soil (*p* < 0.05) (Table 5). For example, the lowest percentage of essential oil in *T. pulegioides* and the highest amount of manganese in soil was in cluster 1′ of *T. pulegioides* habitats; the lowest amount of essential oil and the highest amount of cobalt in soil was in the habitats of the cluster 3′ (Table 2 and Table 4). The percentage of carvacrol in essential oils positively (*p* < 0.05) correlated with the amount of mobile phosphorus and sulphur but negatively with amount of natrium in soil; the percentage of γ-terpinene also correlated positively (*p* < 0.05) with amount of mobile phosphorus in soil. The highest mean percentages of carvacrol, γ-terpinene in *T. pulegioides* essential oils as well as amounts of mobile phosphorus and sulphur in soils were established in habitats of cluster 3′ (Table 2 and Table 4). Percentages of geraniol and biogenetically related compounds (geranial, nerol and neral) in essential oils (*p* < 0.05) positively correlated with amount of natrium and negatively with amounts of chlorine and sulphur (except geraniol) in soils. The highest percentages of geraniol, geranial, nerol and neral in essential oils and the highest and the lowest mean amounts of natrium and chlorine in soil, respectively, were established in *T. pulegioides* habitats belonging to cluster 1′ (Table 2 and Table 4). Also, geraniol and geranial in essential oils correlated negatively (*p* < 0.05) with amount of humus, meanwhile amounts of nerol and neral—negatively with amount of calcium in soil (Table 5). There the amounts of geraniol and biogenetically related compounds in essential oils were the highest, the amount of humus—the lowest in *T. pulegioides* habitats of cluster 1′ (Table 2 and Table 3).

Chemical composition of soil can affect not only percentages of chemotype defined chemical compounds but also percentages of other chemical compounds in essential oils of *T. pulegioides*, for example, percentage β-caryophyllene positively (*p* < 0.05) correlated with amounts of aluminium, copper, iron, potassium, phosphorus, meanwhile percentage of myrcene—negatively (*p* < 0.05) with amounts of potassium and titanium in soils (Table 5). 

### 2.3. Climatic Conditions of Lithuania and Their Effect on Composition of Thymus pulegioides Essential Oils 

There are four climatic districts with 10 climatic sub-districs in the territory of Lithuania: Pajūris (Kuršių Nerija, Pajūris and Pajūris Lowland climatic sub-district), Žemaitija height (Žemaičiai and Venta climatic sub-district), Middle of Lithuania (Mūša-Nevėžis and the Nemumas Lowland climatic sub-district), heights of Lithuania Southeast (Aukštaitija, Dzūkija and Sūduva climatic sub-district). These climatic sub-districts differ by temperature, rainfall, sunshine duration, photosynthetically active solar radiation (Table 6). The one-way analysis of variance (one-way ANOVA) showed that Lithuania climatic sub-districts differ by the sum of temperature and sum of sunshine duration in April–July. Tukey’s post-hoc test showed that the Nemunas Lowland climatic sub-district differs from Žemaičiai, Venta and Pajūris climatic sub-districts by the sum of temperature in April–July. The Nemunas Lowland climatic sub-district distinguishe by the highest, meanwhile Venta, Žemaičiai and Pajūris climatic sub-districts—by lower sum temperature in April–July. Tukey’s post-hoc test also showed that Dzūkija climatic sub-district differs from Žemaičiai, Pajūris and Pajūris Lowland climatic sub-districts by the sum of sunshine duration in April–July. The sum of sunshine duration was the lowest in Dzūkija climatic-subdistrict, meanwhile this climatic factor was higher in Žemaičiai, Pajūris and Pajūris Lowland climatic sub-districts. Rainfall was the most variable climatic factor in Lithuania (Table 6). 

*T. pulegioides* growing in the habitats of Pajūris climatic sub-district accumulated the highest amounts, growing in the habitats of Venta climatic sub-district—the lowest amounts of essential oil (Figure 3). However, the habitats of climatic sub-districts did not differ significantly according to amounts of essential oil in *T. pulegioides* (F = 1.45, *p* > 0.05).

**Figure 4:**
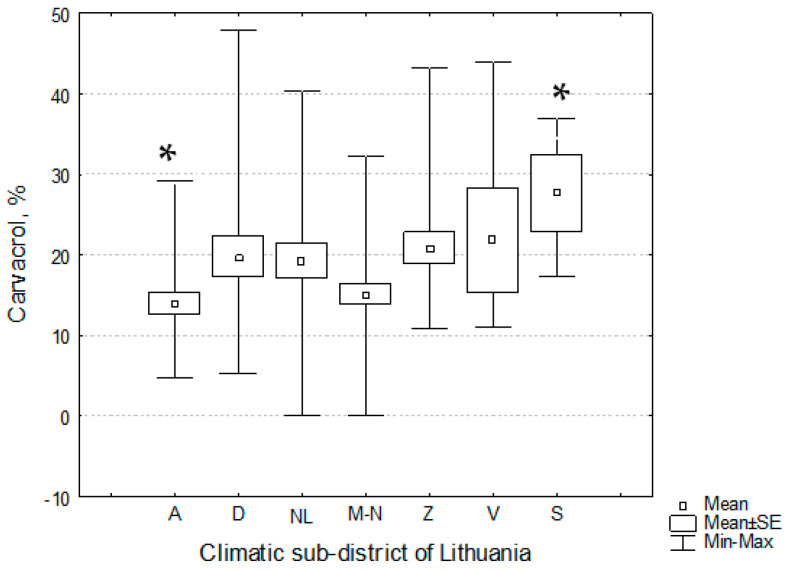
Distribution of the amount of carvacrol in the essential oils of *Thymus pulegioides* habitats in different climatic sub-districts of Lithuania. Explication: A—Aukštaitija climatic sub-district, D—Dzūkija, M-N—Mūša Nevėžis, NL—the Nemunas Lowland, S—Sūduva, V—Venta, Z—Žemaičiai. * denote statistically significant (*p* < 0.05) differences.

Carvacrol dominated in seven sub-districts of Lithuania (Table 7). The one-way analysis of variance and Tukey’s post-hoc test demonstrated that the habitats of *T. pulegioides* in Aukštaitija and Sūduva climatic sub-districts significantly (*p* < 0.05) differed by the percentage of carvacrol in the essential oil; the lowest and the highest mean percentages of carvacrol were established in Aukštaitija and Sūduva sub-districts, respectively (Table 7, Figure 4).

**Figure 5:**
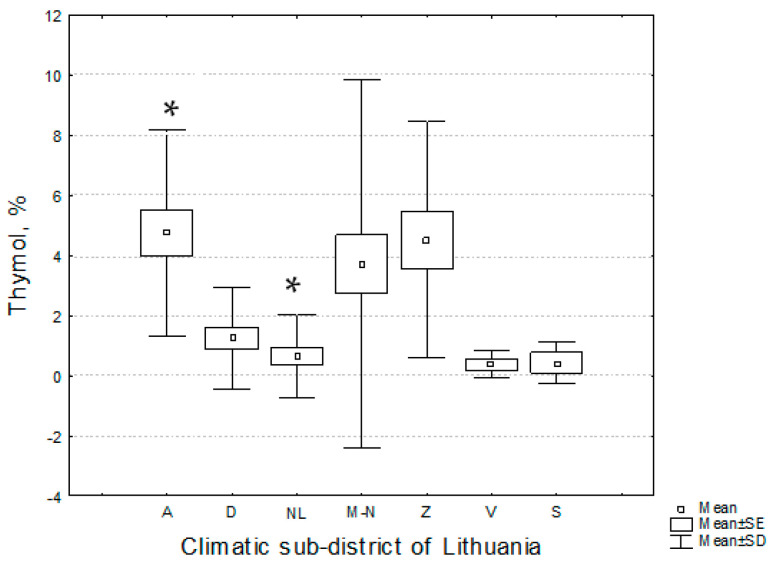
Distribution of the percentage of thymol in the essential oils of *Thymus pulegioides* habitats in different climatic sub-districts of Lithuania. Explication: A—Aukštaitija climatic sub-district, D—Dzūkija, M-N—Mūša Nevėžis, NL—the Nemunas Lowland, S—Sūduva, V—Venta, Z—Žemaičiai. * denote statistically significant (*p* < 0.05) differences.

The one-way analysis of variance and Tukey’s post-hoc test demonstrated that *T. pulegioides* habitats in Aukštaitija (with fixed the highest percentage of thymol in the essential oil) and in the Nemunas Lowland (with the lowest percentage of thymol) climatic sub-districts significantly (*p* < 0.05) differed by the percentage of thymol in the essential oil (Table 7, Figure 5). The percentage of thymol and thymol methyl ether had high variation: the highest variation of thymol and thymol methyl ether was in the Nemunas Lowland and Mūša-Nevėžis climatic sub-district, respectively. 

The variation of percentage of carvacrol and carvacrol methyl ether was lower in comparison with above mentioned chemical compounds (Table 7). *T. pulegioides* habitats of Venta climatic sub-district characterized by the lowest percentages of p-cymene and γ-terpinene and significantly (*p* < 0.05) differed from habitats in Aukštaitija and Mūša-Nevėžis climatic sub-districts according to percentages of these precursors in essential oil. The mean percentage of p-cymene in habitats of Venta climatic sub-district did not reach even 1%. The lowest percentage of γ-terpinene also was established in Venta climatic sub-district. *T. pulegioides* habitats of Žemaičiai climatic sub-district significantly (*p* < 0.05) differed from Venta, Aukštaitija and Mūša-Nevėžis climatic sub-districts according to the percentage of γ-terpinene in the essential oil of *T. pulegioides* (Table 7). 

**Figure 6:**
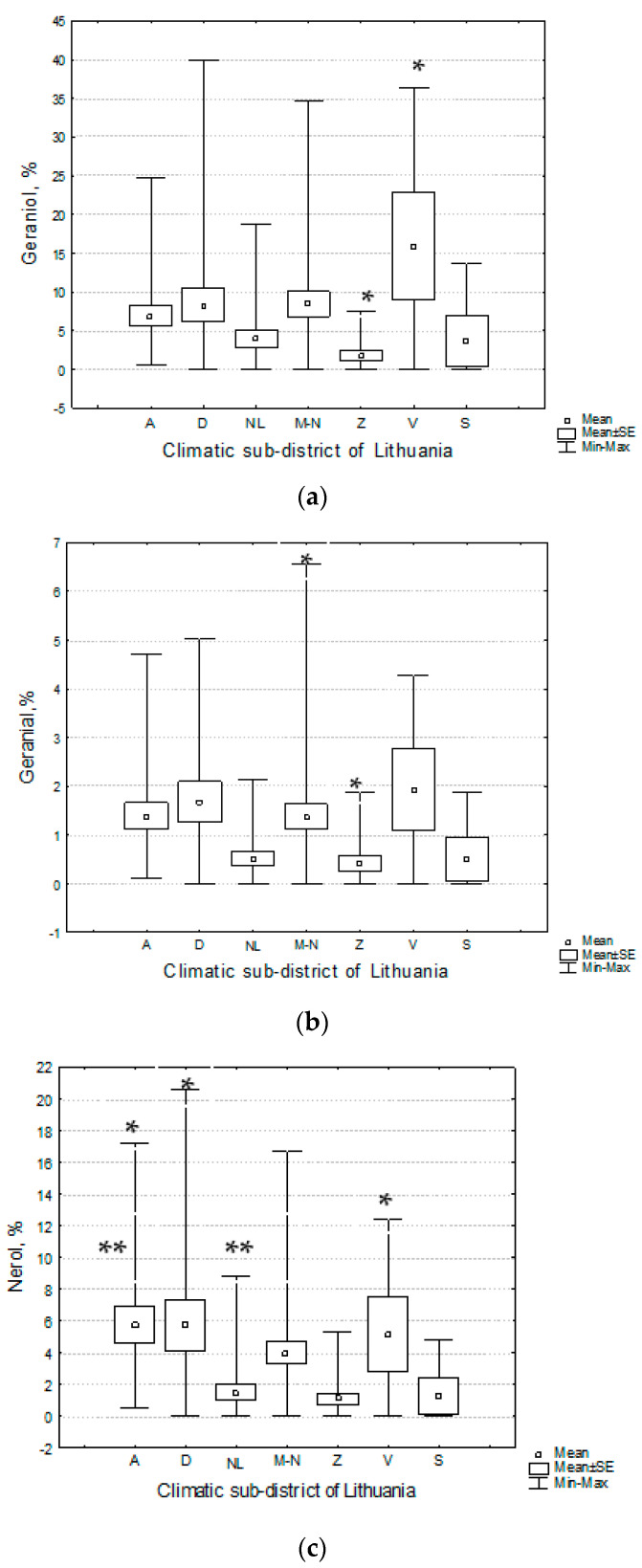

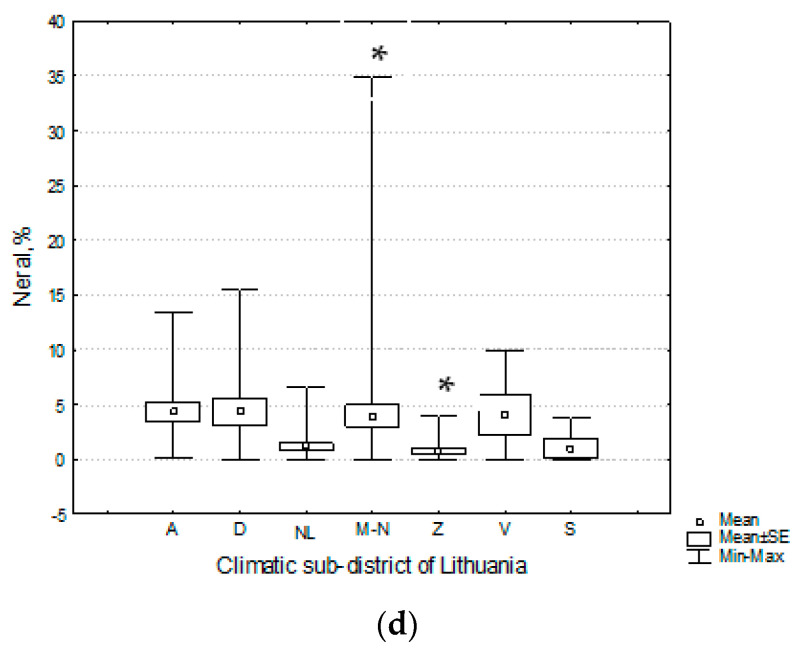
Distribution of the amount of geraniol (**a**), geranial (**b**), nerol (**c**), neral (**d**) in the essential oils of *Thymus pulegioides* habitats in different climatic sub-districts of Lithuania. Explication: A—Aukštaitija climatic sub-district, D—Dzūkija, M-N—Mūša Nevėžis, NL—the Nemunas Lowland, S—Sūduva, V—Venta, Z—Žemaičiai. * denote statistically significant (*p* < 0.05) differences. ** denote statistically significant (*p* < 0.05) differences between habitats of Aukštaitija and the Nemunas Lowland climatic sub-districts by amount of nerol.

The highest mean percentages of geraniol and geranial were established in Venta climatic sub-district, the highest of nerol and neral—in Aukštaitija climatic sub-district; meanwhile the lowest percentages of these geraniol chemotype characterising chemical compounds were established in samples of plant raw material collected in Žemaičiai climatic sub-district. The one-way ANOVA and Tukey’s post-hoc test demonstrated that *T. pulegioides* habitats in Žemaičiai and Venta climatic sub-districts significantly (*p* < 0.05) differed by percentage of geraniol, the habitats in Mūša-Nevėžis and Žemaičiai climatic sub-districts—by percentages of geranial and neral. *T. pulegioides* habitats in Venta climatic sub-district significantly (*p* < 0.05) differed from habitats in Dzūkija and Aukštaitija climatic-subdistricts by percentage of nerol; habitats in Aukštaitija climatic sub-district differed significantly (*p* < 0.05) from habitats in the Nemunas Lowland climatic sub-district also by percentage of this chemical compound (Table 7, Figure 6). 

Significant differences of percentages of linalool and α-terpinyl acetate (these chemical compounds define *T. pulegioides* linalool and α-terpinyl acetate chemotypes, respectively) were not established between *T. pulegioides* habitats of different climatic sub-districts (Table 7). *T. pulegioides* habitats in the different climatic sub-district differed also by chemical compounds of essential oil what non define chemotypes, for example, by percentage of β-bisabolene, caryophyllene oxide, α-terpinene, cis-β-guaiene (Table 7). 

### 2.4. Influence of Edaphic and Climatic Factors on Prevalention of Geraniol and Carvacrol Chemotypes of T. pulegioides 

**Figure 7:**
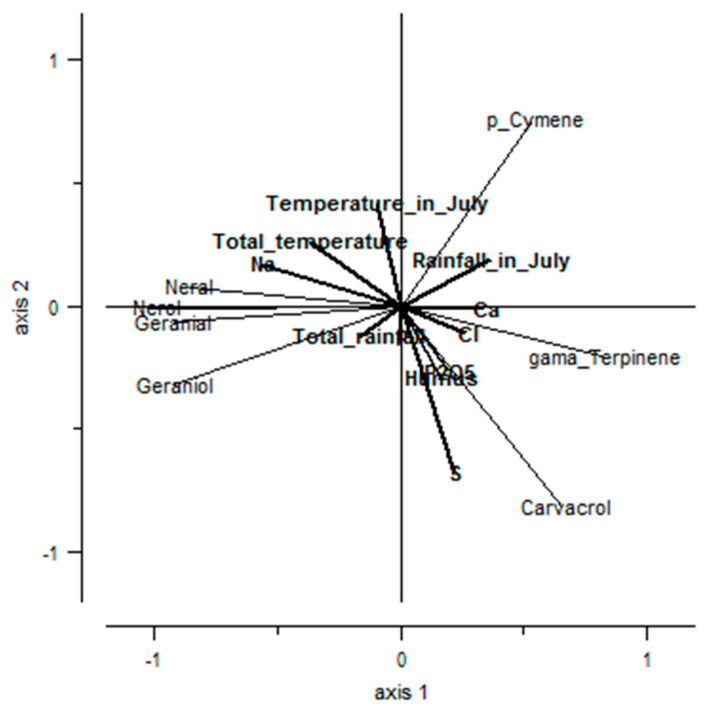
Dependence of the amounts of *Thymus pulegioides* geraniol and carvacrol chemotype-determining compounds in essential oils on edaphic and climatic factors (redundancy analysis).

The redundancy analysis (RDA) showed that the studied edaphic and climatic factors could explain the prevalence of 17% of carvacrol and geraniol chemotypes-determining compounds of *T. pulegioides* (Figure 7). Thymol, linalool and α-terpinyl acetate chemotype-determining compounds were not included in this analysis since the influence of edaphic and climatic factors on the prevalence of these compounds was not established. It can be assumed that genetic factors have greater influence on the prevalence of carvacrol and geraniol chemotypes of *T. pulegioides*. Edaphic factors had a stronger influence on the prevalence of *T. pulegioides* carvacrol and geraniol chemotypes (edaphic factors explain 9% prevalence, climatic conditions 8% prevalence of carvacrol and geraniol chemotypes) than climatic conditions. This analysis also confirmed that higher content of sulphur and mobile phosphorus (P_2_O_5_) in the soil had positive, whereas that of sodium had a negative influence on the prevalence of *T. pulegioides* carvacrol chemotype, and that geraniol chemotype can be detected more often with higher content of natrium, less frequently with higher content of chlorine in the soil. The positive relationship between the content of geraniol and the total temperature in July and April–July, the prevalention of *T. pulegioides* geraniol chemotype is positively influenced by a higher temperature. The positive relationship between carvacrol, p-cymene and γ-terpinene and rainfall in July were detected also (Figure 7). 

## 3. Discussion

Chemotypes of genus *Thymus* are determinated genetically, but chemical composition of soil, climatic conditions, altitude can influence quantitative and qualitative composition of essential oils [15,18]. Edaphic and climatic conditions in the territory of Lithuania are not equal. The central territory of Lithuania characteristic by higher amounts of humus, mobile phosphorus, mobile potassium, calcium, and magnesium in soil, the western part of Lithuania—by more natrium and iron in soils and higher soil acidity [24,25]. The marine climate is more pronounced in the western, meanwhile the continental climate—in the eastern part of Lithuania, therefore the climatic sub-districts, distinguished in Lithuania, differ by the average annual temperature, the precipitation per year, the duration of snow preservation [23]. The one-way analysis of climatic conditions in 2006–2016 period also showed that climatic sub-districts differ significantly by sum temperature and sum sunshine duration in April–July: the highest sunshine duration was in the west of Lithuania (Žemaičiai, Pajūris and Pajūris Lowland climatic sub-districts), the highest sum temperature—in the Nemunas Lowland climatic sub-district of the central territory of Lithuania (Table 6). Therefore, differences of edaphic and climatic conditions in Lithuania can influence the composition of essential oils of there growing *T. pulegioides*, as well as the distribution of *T. pulegioides* chemotypes in space. 

Studies demonstrated that all territory of Lithuania is suitable for *T. pulegioides*, and the amount of essential oil in habitats of *T. pulegioides* varied from 0.20% to 1.32%. Literature data suggest that amount of essential oils of *T. pulegioides* growing in other European countries also can vary in the wide ranges: in Romania amount of essential oil varied from 0.7% to 1.1% [27], in Croatia—from 0.6% to 1.31% [11], in Portugal—1.8%, [28], in Kosovo—1.58% [29]. The analysis showed that higher amount of humus in soil positively, higher amounts of manganese and cobalt—negatively influenced the amount of essential oil in raw material collected in habitats of *T. pulegioides* (Table 2, Table 3, Table 4 and Table 5). Humus is the main component of soil organic matter that improves the fertility, physical, chemical and biological features of soil, the process of uptake of the macro- and microelements, as well as the water regime, reduces abiotic stress [30], therefore, all this can promote not only the growth of plants, but also the accumulation of secondary metabolites in them. For example, essential oil yield of *Lavandula latifolia* (Lamiaceae) was higher in soils, in which was more organic matter [31]. 

Carvacrol was the most abundant and frequent chemical compound and was found in investigated essential oils samples of *T. pulegioides* collected in all habitats (except one habitat only). It allows us to assume that *T. pulegioides* carvacrol chemotype is dominating chemotype in all territory of Lithuania. After analysing of essential oil samples, a low prevalence of carvacrol isomer thymol was determined in Lithuania: the cluster analysis showed that it was dominated only in 17 habitats of *T. pulegioides* (Table 2, Figure 2). Therefore, the thymol chemotype of *T. pulegioides* is rarer in Lithuania compared to the carvacrol chemotype. Previous investigations of *T. pulegioides* in Vilnius district (Lithuania) also showed the domination of carvacrol chemotype in habitats [32,33,34,35]. However, both phenolic (carvacrol and thymol) chemotypes of *T. pulegioides* (and other species of genus *Thymus*) are very frequent and/or dominant in Europe: for example, the carvacrol chemotype dominated in Romania (carvacrol amounted from 50.5% to 62.6% of essential oil) [27], the thymol chemotype—in investigated regions of Portugal and Italy [1,28], both phenolic chemotypes—in Norway and Yugoslavia [36,37]. Percentage of carvacrol correlated positively (*p* < 0.05) with the amount of mobile phosphorus (this relationship was also characteristic of carvacrol precursor γ-terpinene) and sulphur but negatively (*p* < 0.05) with the amount of natrium in soil (Table 2, Table 3, Table 4 and Table 5, Figure 6). Previous studies also indicated that the increase of sulphur in soil can positively influence the accumulation of carvacrol in *T. pulegioides* and *Thymus pannonicus* [38,39], as well as in *Sarureja montana* (Lamiaceae) [40]. Therefore, very likely that the carvacrol chemotype of *T. pulegioides* may be more common in soils with the high amount of mobile phosphorus, as well as with higher and lower amount of sulphur and natrium, respectively. Also, carvacrol dominated in essential oils samples collected in *T. pulegioides* habitats of Sūduva climatic sub-district where was the most precipitation in June and July (in the blooming period of *T. pulegioides*) (Table 6), which may indicate a greater preference of carvacrol chemotype for wetter climate. However, literature data about a relationship of amount of carvacrol with humidity are different: was established that lower amount of moisture reduces the amount of carvacrol in *Thymus numidicus* [41] but increases in *T. vulgaris* [18,42]. The largest percentage of thymol was established in Aukštaitija climatic sub-district, i.e., in the eastern part of Lithuania (Table 7), where the temperature in January is the lowest compared with other climatic sub-districts of country [23]. Therefore, the thymol chemotype of *T. pulegioides* may be more adapted to the continental climate. Literature data also suggest that the individuals of thymol chemotype are more resistant to the cold [43,44]. 

Geraniol, though the percentage of which was 2.7 times lower than the percentage of carvacrol, was the second common chemical compound in the investigated samples of essential oils of *T. pulegioides* (Table 1). Assuming that the geraniol chemotype was prevailing in the habitats, in which geraniol was the main chemical compound of essential oils, this chemotype could dominate in ¼ of investigated habitats of *T. pulegioides* (Table 2, Figure 3). Results showed that geraniol was more common in the eastern and the central Lithuania with the more continental climate because there temperature in January is lower [23], the sum temperature in April–July higher (except Sūduva climatic sub-district) compared to other parts of Lithuania (Table 6). Previous studies also showed the positive effect of temperature on geraniol percentage in *Thymus pulegioides* plants of geraniol chemotype [22]. Individuals of *T. vulgaris* geraniol chemotype were also more tolerant to low temperature and temperature fluctuation in winter [42,43,44,45]. All these facts may explain the greater attachment of the geraniol chemotype to a continental climate. Percentage of geraniol also was higher in the essential oil samples collected from habitats, in soils of which was found higher amount of natrium and lower amount of humus, sulphur and chlorine (Figure 6). Therefore, geraniol chemotype of *T. pulegioides* may be less demanding on soil fertility. Although, geraniol in *Rosa damascene*, in the flower essential oil of which this monoterpene alcohol is one of main compounds, correlated positively with silt, negatively—with sand in soil [46]. 

Linalool and α-terpinyl acetate were established in the samples of essential oils from the most *T. pulegioides* habitats, but on average they constituted less than 2% of essential oils (Table 1). Therefore, linalool and α-terpinyl acetate chemotypes are not common in the territory of Lithuania. The cluster analysis showed that α-terpinyl acetate was dominated only in three habitats of *T. pulegioides* and linalool—in two habitats (Table 2, Figure 2). There is evidence that the linalool chemotype of *T. pulegioides* dominates in some one habitats in Poland and Slovakia [2,47], α-terpinyl acetate—in habitats of subalpine locations in France [4]. Found max percentages of linalool and α-terpinyl acetate were 1.2–1.4 time higher than the max percentages of carvacrol and geraniol (Table 1), i.e., habitats with the domination of linalool or α-terpinyl acetate chemotypes are very rare. Therefore, it was not possible to determine a potential attachment of linalool and α-terpinyl acetate chemotypes of *T. pulegioides* to some climatic conditions or chemical composition of soil.

Edaphic and climatic conditions can differently influence not only on essential oil yield and chemical composition of species: study showed that these environmental factors may effect on the prevalence of different chemotypes of same species in space. 

## 4. Materials and Methods

### 4.1. Plant Material 

One hundred and thirty-one different habitats of *T. pulegioides* were investigated in Lithuania (Figure 7). The study of the habitats was carried out in all climatic sub-districts of Lithuania: in Aukštaitija climatic sub-district were studied 21 habitats of *T. pulegioides*, in Dzūkija—20, in Mūša-Nevėžis—40, in the Nemunas Lowland—22, in Sūduva—4, in Venta—5, and in Žemaičiai climatic sub-district—17 habitats. One *T. pulegioides* habitat was found and investigated in Pajūris and Pajūris Lowland climatic sub-districts.

The aerial parts of *T. pulegioides* were collected for plant raw material in full flowering period (in July). The plant raw material was collected in each habitat in the following way: same selected weight of aerial part of *T. pulegioides* was cut from each individual plant growing in the habitat and theses all aerial parts mixed (therefore, one mix (sample) represented one investigated habitat); the weight of plant raw material was selected depending on habitat abundance and/or size of individual plants in the habitat: 10 g from each *T. pulegioides* individual plant was cut in abundant and big habitats, 30–50 g from each *T. pulegioides* individual plant—in small habitats. The plant raw material collected from each habitat was dried separately at room temperature.

### 4.2. Isolation and Investigation of Essential Oils

The essential oil from each plant raw material sample (as was mentioned above, one sample represented one habitat) was isolated separately by hydrodistillation in at least three replicates in the Clevenger apparatus [48]; each hydrodistillation was carried out two hours. All distillation replicates of essential oils, extracted from a single plant raw material sample, were pooled into a single vial (therefore, one essential oil sample represented one habitat). 

Essential oils solutions of 1% were prepared in the mixture of diethyl ether and n-pentane (1:1) for further investigations. The identification of the main compounds of essential oils was based on an GC-2010 Plus instrument equipped (Shimadzu) with a GC-QP 2010 Plus (Shimadzu) series mass selective detector in the electron impact ionisation mode at 70 eV. Separation of compounds was performed on fused silica (100% dimethyl polysiloxane) column (30 m × 0.25 mm ID × 0.25 µm film thickness) (Restek, Bellefonte, PA, USA), splitless injection; helium as carrier gas at a flow rate of 1.6 mL/min, injector and detector temperatures 250 °C. GC oven temperature was programmed as follows: initial temperature of 50 °C (isothermal for 7 min) was increased to 250 °C at the rate of 4 °C/min to (isothermal for 5 min) and further increased at the rate of 30 °C/min to 300 °C, the final temperature kept for 2 min. Identification of the investigated compounds was based on the comparison of retention indices (RIs) [48], computer mass spectra library (NBS75K) and analytical standards of these (Sigma-Aldrich, Praha, Czech Republic). The retention indices were determined relative to the retention times of a series of n-alkanes (C7–C30) with linear interpolation. The quantitative analysis of main compounds was carried out using a FOCUS GC (Thermo Scientific, Waltham, MA, USA) gas chromatograph with a flame ionisation detector (FID) on the silica capillary column TR-5MS (30 m × 0.25 mm ID × 0.25 μm film thickness) (Thermo Electron Corporation, Waltham, MA, USA) under the same chromatographic conditions. The percentage of the investigated compounds were recalculated according to the areas of the FID chromatographic peaks assuming that all constituents of the essential oil comprise 100%. 

### 4.3. Collection and Investigation of Soil

Each sample of topsoil was prepared in the following way: 5–9 subsamples (subject to the area of the habitat; each subsample ~100 g) were taken from the depth of 10–15 cm (from plant rhizosphere) by the envelope principle with the distance of 1 m from the central point of habitat and mixed (homogenized).The content of humus in the samples of topsoil was estimated by oxidation method, using potassium bichromate and sulphur acid solution, the content of mobile potassium (K_2_O) and mobile phosphorus (P_2_O_5_)—by flame photometry using 0.2 M HCl solution, the soil pH—electrometrically using 1 M KCl solution. The elements of soil (Al, Ca, Cu, Fe, K, Mg, Mn, Na, P, Co, S, Si, Ti, Zn, Cl) were estimated the by energy-dispersive X-ray fluorescence analysis (EDXRF equipment SPECTRO SCEPOS). 

### 4.4. Analysis of Meteorological Data

The meteorological data (temperature (°C), rainfall (mm), photosynthetically active solar radiation (PAR) (Mj/m^2^) and sunshine duration (h)) were obtained from the meteorological bulletins (2006–2016) of the nearest station of meteorology of the Lithuanian Hydrometeorological Service under the Ministry of Environment. The photosynthetically active solar radiation was calculated by multiplying the total solar radiation a factor of 0.52 [23]. For the analysis we used the mean values of these factors (June, July is a period of *T. pulegioides* blooming and Σ_April–July_ is a period from the beginning of vegetation to flowering) obtained in 2006–2016. The meteorological data were analyzed in nineteen meteorological stations: Biržai, Dotnuva, Dūkštas, Kaunas, Klaipėda, Kybartai, Laukuva, Lazdijai, Nida, Panevežys, Raseiniai, Šiauliai, Šilutė, Telšiai, Ukmergė, Varėna, Vilnius and Mažeikiai. The averages of the above-mentioned meteorological factors were calculated in case several meteorological stations belonged to one climatic sub-district. 

### 4.5. Statistical Analysis

The calculation of means, standard deviations (SD), coefficients of variation (CV), determination of minimum and maximum values was carried out. Cluster analysis by Ward’s method was performed by grouping *T. pulegioides* habitats based on chemotype-determining compounds (carvacrol, thymol, p-cymene, γ-terpinene, carvacrol methyl ether, thymol methyl ether, geraniol, geranial, nerol, neral, linalool and α-terpinyl acetate). Spearman’s rank correlation coefficients (r) were used for analysis of influence for soil chemical composition on *T. pulegioides* essential oil yield and composition. One-way ANOVA analysis was used for accession if *T. pulegioides* habitats in climatic sub-districts differ according to quantitative and qualitative composition of essential oils and prevalence of chemotypes. One-way ANOVA analysis also was used for accession if Lithuanian climatic sub-districts differ according to temperature, rainfall, photosynthetically active solar radiation and sunshine duration. Tukey’s post-hoc criterion was used for accession of differences between *T. pulegioides* habitats in different climatic sub-districts or between Lithuanian climatic sub-districts. Redundancy analysis (RDA) was performed to find out what part of chemotype-determining compounds could be explained by the environmental factors (edaphic and climatic) and what (edaphic or climatic factors) had greater influence on the prevalence of these chemical compounds. 

## 5. Conclusions

Carvacrol was the most abundant and frequent and geraniol was the second chemical compound in the essential oil of *T. pulegioides* growing wild in Lithuania. *T. pulegioides* carvacrol chemotype can be more common with higher amounts of mobile phosphorus and sulphur, lower amount of natrium in soils and wetter climatic condition. *T. pulegioides* geraniol chemotype can be more common with higher amount of natrium, lower amounts of chlorine and sulphur in soils and higher temperature in vegetation period. It was not determined a potential attachment of linalool and α-terpinyl acetate chemotypes of *T. pulegioides* to some climatic conditions or chemical composition of soil.

## Figures and Tables

**Table 1:** Descriptive statistics of chemical compounds (the percentage of which exceeded for leastways in one habitat) of essential oils of *Thymus pulegioides* in investigated habitats (N = 131).

Chemical Compound	Retention Index		Min–Max, %	Mean ± SD, %	CV, %
	Calculated	Literature [26]			
Carvacrol	1308	1298	0.00–48.00	17.66 ± 9.43	53
Thymol	1298	1289	0.00–31.00	3.17 ± 5.11	162
p-Cymene	1029	1020	0.14–38.49	11.81 ± 8.21	70
γ-Terpinene	1053	1054	0.00–42.60	16.90 ± 8.83	52
Geraniol	1237	1249	0.00–39.87	6.57 ± 8.70	132
Geranial	1272	1264	0.00–6.57	1.13 ± 1.44	127
Nerol	1235	1227	0.00–20.57	3.66 ± 4.90	134
Neral	1242	1235	0.00–34.92	3.05 ± 4.73	155
Linalool	1104	1095	0.00–57.75	1.66 ± 6.59	397
α-Terpinyl acetate	1355	1346	0.00–57.50	1.48 ± 6.96	470
Thymol methyl ether	1241	1232	0.00–5.79	0.83 ± 1.14	470
Carvacrol methyl ether	1251	1241	0.00–9.17	3.97 ± 2.22	56
Myrcene	997	988	0.00–4.19	1.48 ± 6.96	39
β-Caryophyllene	1426	1417	0.69–13.66	5.52 ± 1.82	33
β-Bisabolene	1513	1505	0.00–6.66	3.02 ± 1.15	38
Caryophyllene oxside	1591	1582	0.00–5.01	1.16 ± 0.73	63
α-Terpinene	1022	1014	0.00–4.85	1.64 ± 0.85	52
Borneol	1173	1165	0.00–4.65	0.56 ± 0.53	95
Cis-β-Guaiene	1500	1492	0.00–7.14	1.65 ± 0.89	39

**Table 2:** Descriptive statistics of essential oils and percentages of main chemical compounds of chemotypes (carvacrol, thymol, geraniol, linalool, α-terpinyl acetate) and their precursors and biogenetically related compounds in distinguished clusters of *Thymus pulegioides* habitats and habitats no. 11, no. 34, no. 39, no. 99, no. 100 and no. 106.

Cluster or Habitat Number.		Amount of Essential Oil, %	Carvacrol, %	Thymol, %	p-Cymene, %	γ-Terpinene, %	Carvacrol Methyl Ether, %	Thymol Methyl Ether, %	Geraniol, %	Geranial, %	Nerol, %	Neral, %	Linalool %	α-Terpinyl Acetate, %
Cluster 1′ (N = 34)	Mean	0.55	11.24	1.55	4.94	8.86	2.04	0.37	18.56	3.09	9.92	8.63	2.05	4.94
	SD	0.21	5.51	2.46	3.85	6.36	0.99	0.51	8.90	1.44	5.40	6.18	4.94	2.01
	Min	0.23	0.00	0.00	0.39	0.00	0.12	0.00	3.48	0.32	0.88	0.65	0.00	0.00
	Max	1.21	24.28	10.28	12.60	23.85	3.67	2.02	39.87	6.57	20.57	34.92	22.94	9.84
	CV, %	38	49	159	78	72	49	138	48	47	54	72	241	41
Cluster 2′ (N = 17)	Mean	0.64	13.38	12.10	17.61	16.59	4.42	3.19	2.39	0.40	1.43	1.02	0.36	0.27
	SD	0.12	4.12	7.48	7.27	6.59	1.55	1.33	2.85	0.46	1.73	1.35	0.10	0.53
	Min	0.83	7.09	4.50	5.20	0.81	2.23	1.48	0.00	0.00	0.00	0.00	0.16	0.00
	Max	0.65	21.31	31.00	29.55	30.64	6.50	5.79	10.04	1.45	5.66	4.23	0.51	1.84
	CV, %	19	31	62	41	40	35	42	119	115	121	132	28	196
Cluster 3′ (N = 45)	Mean	0.68	26.11	2.09	9.44	23.78	3.89	0.55	1.89	0.29	0.75	0.56	0.56	0.07
	SD	0.22	8.75	2.96	4.41	6.40	1.99	0.63	2.21	0.40	1.13	0.90	0.75	0.24
	Min	0.23	12.10	0.00	1.23	11.96	0.00	0.00	0.00	0.00	0.00	0.00	0.00	0.00
	Max	1.32	48.00	11.11	20.00	42.60	9.06	2.21	7.80	1.40	5.29	4.16	4.41	1.35
	CV, %	32	34	142	47	27	51	115	117	138	151	161	134	343
Cluster 4′ (N = 28)	Mean	0.57	17.89	1.56	20.66	17.49	6.25	0.54	2.94	0.72	2.72	2.06	0.35	0.34
	SD	0.22	5.38	1.72	7.17	5.96	1.40	0.51	2.66	0.60	2.59	1.97	0.22	1.01
	Min	0.20	8.19	0.00	5.73	4.40	3.02	0.00	0.00	0.00	0.00	0.00	0.10	0.00
	Max	1.03	27.10	6.36	38.49	29.47	9.17	2.27	10.04	1.88	9.34	7.18	1.23	4.33
	CV, %	39	30	111	35	34	22	94	90	83	95	96	63	297
Habitat no. 11	Amount	0.39	13.58	2.38	20.99	11.68	6.94	1.46	1.67	0.17	0.65	0.09	14.25	2.34
Habitat no. 34	Amount	1.02	5.65	1.32	6.82	5.87	1.72	0.40	2.92	0.56	1.87	1.43	0.35	43.56
Habitat no. 39	Amount	0.53	5.65	0.00	20.70	6.44	5.77	0.05	2.08	0.12	0.07	0.00	6.51	30.77
Habitat no. 99	Amount	0.54	0.06	0.00	0.14	0.48	0.04	0.02	12.35	1.12	3.52	2.81	0.29	57.50
Habitat no. 106	Amount	0.77	6.39	0.00	5.38	5.85	1.88	0.53	6.36	1.32	4.78	3.87	40.37	3.99
Habitat no. 100	Amount	0.53	3.48	0.27	1.31	1.57	0.58	0.00	4.01	0.38	1.01	0.90	57.75	6.01

**Table 3:** Descriptive statistics of pH_KCl_, humus, mobile potassium (K_2_O), mobile phosphorus (P_2_O_5_) in distinguished clusters of *Thymus pulegioides* habitats and habitats no. 11, no. 34, no. 39, no. 99, no. 100 and no. 106.

		pH_KCl_	Humus, %	K_2_O, mg/kg	P_2_O_5_, mg/kg
N = 131	Mean ± SD	7.37 ± 0.57	2.70 ± 1.09	121.90 ± 63.97	127.28 ± 95.16
	Min–Max	5.1–8.3	0.9–7.2	35.00–368.00	22.00–680.00
	CV, %	8	40	52	74
Cluster 1′ (N = 34)	Mean ± SD	7.46 ± 0.56	2.47 ± 0.97	113.21 ± 46.38	123.41 ± 84.07
	Min–Max	6.0–8.2	1.0–5.2	44.00–236.00	24.00–411.00
	CV, %	8	39	41	68
Cluster 2′ (N = 17)	Mean ± SD	7.27 ± 0.40	2.57 ± 0.99	99.29 ± 36.06	118.35 ± 106.07
	Min–Max	5.1–8.1	1.0–4.4	44.00–149.00	23.00–424.00
	CV, %	6	38	36	90
Cluster 3′ (N = 45)	Mean ± SD	7.40 ± 0.53	2.72 ± 0.99	125.89 ± 67.93	135.36 ± 80.27
	Min–Max	5.1–8.3	1.0–4.7	35.00–350.00	25.00–457.00
	CV, %	7	36	54	59
Cluster 4′ (N = 28)	Mean ± SD	7.34 ± 0.62	2.78 ± 1.40	132.21 ± 72.57	117.61 ± 125.45
	Min–Max	5.9–8.2	0.9–7.2	50.00–356.00	22.00–680.00
	CV, %	8	50	55.0	107.0
Habitat no. 11	Amount	6.9	4.0	215.0	81.0
Habitat no. 34	Amount	7.6	3.9	163.0	186.0
Habitat no. 39	Amount	8.0	1.4	79.0	65.0
Habitat no. 99	Amount	7.4	5.0	368.00	313.0
Habitat no. 100	Amount	6.1	2.0	64.0	45.0
Habitat no. 106	Amount	7.6	2. 4	87.0	177.0

**Table 4:** Descriptive statistics of chemical elements of soil in distinguished clusters of *Thymus pulegioides* habitats and habitats no. 11, no. 34, no. 39, no. 99, no. 100 and no. 106.

Cluster or Habitat Number		Al	Ca	Cu	Fe	K	Mg	Mn	Na	P	S	Si	Ti	Zn	Co	Cl
N = 131	Mean	26,505.50	24,315.50	8.15	11,808.30	17,743.70	7191.4	350.50	5894.30	477.90	226.1	380,643.1	1652.8	40.10	4.1	385.30
	SD	10,151.59	15,759.48	3.64	5436.02	3856.66	4685.60	159.17	1096.45	484.86	134.31	39,914.34	817.53	13.72	2.39	207.4
	Min	11,450	1894	2.6	4489	11,007	627	150	3055	106	15	278,411	436	20	0.4	83
	Max	73,099	64,606	20.1	34,261	31,381	19,095	1501	9114	4997	657	450,223	4547	95	10.8	1862
	CV, %	38	38	45	46	22	65	45	19	101	59	10	49	34	58	54
Cluster 1′ (N = 34)	Mean	24,751.20	24,038.20	8.01	11,086.50	17,676.0	7439.00	364.1	6175.70	438.20	208.80	380,739.20	1725.80	38.70	4.30	321.9
	SD	7303.22	16,944.48	3.41	4628.63	3230.52	5354.14	164.8	1120.90	301.35	141.88	38,176.68	853.25	14.53	2.08	96.15
	Min	12,122	3121	3.8	4649	11,924	1134	168	4282	113	15	278,411	436	20	0.4	135
	Max	48,441	57,963	20.1	30,803	27,362	19,095	1090	9114	1876	657	435,162	3972	89	8.8	506
	CV, %	30	70	43	42	18	72	45	18	69	46	10	49	38	48	30
Cluster 2′ (N = 17)	Mean	24,082.04	23,161.10	7.17	10,241.46	16,418.63	5984.63	342.63	6019.8	665.9	185.5	390,674.6	1355.7	35.8	3.80	403.2
	SD	7278.88	14,648.68	3.31	3744.89	2825.31	4213.87	104.07	1043.34	1130.87	136.64	36,630.22	536.97	9.67	2.21	126.64
	Min	11,450	2766	3.4	5213	11,531	803	197	4576	106	30	336,270	517	22	0.4	83
	Max	41,763	41,793	13.8	18,489	22,014	15,052	558	8074	4997	556	450,223	2337	52	8.4	587
	CV, %	30	63	46	37	17	70	30	17	170	74	9	40	27	58	31
Cluster 3′ (N = 7)	Mean	25,709.90	27,069.38	7.89	11,690.32	17,401.04	7771.16	344.70	5827.50	444,5	257.30	376,922.30	1601.77	38.49	3.73	428.35
	SD	9883.03	14,518.34	3.61	5396.30	4055.52	4972.84	198.20	948.68	200.24	130.56	37,250.70	830.40	9.48	2.67	289.67
	Min	14,228	1894	2.60	4489	11,007	627	150	3440	149	63	299,097	589	24	0.4	168
	Max	59,507	53,962	17.3	26,044	27,337	18,841	1501	7854	1014	628	449,804	3477	60	10.7	1863
	CV, %	38	54	46	46	23	64	55	16	45	51	10	52	25	72	68
Cluster 4′ (N = 28)	Mean	30,550.66	22,911.60	8.76	13,536.78	18,696.45	7039.30	355.15	5661.37	395.18	199.21	390,312.90	1777.20	42.20	4.80	356.5
	SD	14,993.53	17,213.48	4.17	7147.57	4820.92	4310.13	130.08	1240.65	189.48	124.36	37,649.37	937.97	16.52	2.6	114.24
	Min	11,450	2176	20.0	5263	11,395	710	197	3055	128	17	313,391	477	21	0.9	129
	Max	73,099	64,606	3.5	34,961	31,381	18,587	791	7908	964	488	442,443	4547	82	10.8	559
	CV, %	49	75	48	53	26	61	37	22	48	62	10	53	39	54	32
Habitat no. 11	Amount	38,652	9196	10	17,544	22,736	6598	348	5453	434	302	380,454	2170	42	5.5	400
Habitat no. 34	Amount	28,422	22,692	9	12,828	20,796	6967	377	5439	540	292	376,059	1865	41	2.9	541
Habitat no. 39	Amount	14,669	56,164	7	6607	12,782	11,473	239	6239	212	96	357,185	493	25	2.8	523
Habitat no. 99	Amount	26,867	36,441	15	15,269	19,967	10,414	396	3367	2331	415	331,517	2203	69	4.2	498
Habitat no. 100	Amount	20,319	3788	3	6304	15,705	1490	201	6776	187	76	432,282	1025	27	2.1	142
Habitat no. 106	Amount	22,644	8964	6	10,211	17,308	3309	263	6347	315	164	407,441	1528	53	3.3	195

**Table 5:** Spearman’s rank correlations between chemical characteristics (amount of essential oil and percentages of main chemical compounds) of essential oil of *Thymus pulegioides* and some topsoil characteristics (humus, soil pH, mobile potassium (K_2_O) mobile phosphorus (P_2_O_5_) and 15 chemical elements. Chemotypes define chemical compounds, precursors, biogenetically related compounds and statistically significant correlation coefficients are bold.

Chemical Characteristics	Humus, %	pH_KCl_	K_2_O, mg/kg	P_2_O_5_, mg/kg	Chemical Elements, mg/kg														
					Al	Ca	Cu	Fe	K	Mg	Mn	Na	P	Co	S	Si	Ti	Zn	Cl
Amount of essential oil (%)	**0.18**	0.14	0.02	0.15	−0.16	0.13	−0.09	−0.16	−0.14	−0.02	**−0.22**	−0.06	0.08	**−0.32**	0.00	0.04	−0.12	−0.04	−0.02
**Carvacrol**	0.10	0.01	−0.08	**0.18**	0.08	0.13	0.09	0.13	0.09	0.14	−0.03	**−0.20**	0.07	−0.08	**0.27**	−0.16	0.13	0.08	0.00
**Thymol**	0.04	−0.14	0.15	0.12	−0.01	−0.05	−0.11	−0.08	−0.07	−0.07	−0.03	−0.04	0.16	−0.06	−0.12	0.12	−0.13	−0.12	0.10
**p-Cymene**	−0.12	0.02	0.01	−0.16	0.00	0.06	−0.02	−0.02	−0.03	−0.05	−0.10	−0.07	**−0.18**	0.02	**−0.20**	0.00	−0.11	−0.10	0.13
**γ-Terpinene**	0.07	−0.04	0.01	**0.23**	−0.06	0.04	−0.10	−0.11	−0.12	−0.15	−0.12	0.08	0.02	−0.14	0.13	0.02	−0.09	−0.04	−0.06
**Geraniol**	**−0.19**	0.05	−0.02	0.04	−0.13	−0.10	−0.12	−0.11	−0.05	−0.09	−0.02	**0.18**	0.04	0.07	−0.16	0.14	0.02	0.08	**−0.22**
**Geranial**	**−0.20**	0.03	−0.03	−0.02	−0.11	−0.13	−0.12	−0.12	−0.04	−0.10	0.00	**0.19**	−0.06	0.14	**−0.23**	0.17	−0.02	−0.09	**−0.20**
**Nerol**	−0.16	−0.05	−0.05	−0.06	−0.10	**−0.22**	−0.10	−0.12	−0.04	−0.16	0.03	**0.22**	0.06	0.15	**−0.25**	**0.23**	−0.03	−0.01	**−0.20**
**Neral**	−0.17	−0.05	−0.03	−0.05	−0.09	**−0.19**	0.10	0.11	−0.03	−0.14	0.03	**0.19**	0.06	0.15	**−0.24**	0.20	−0.02	−0.09	**−0.20**
**Linalool**	0.03	−0.03	0.05	0.16	−0.01	0.02	0.08	0.01	−0.02	0.04	−0.06	0.14	0.06	−0.04	0.08	−0.02	−0.06	−0.02	0.09
**α-Terpinyl acetate**	−0.10	0.00	0.08	0.14	0.09	0.07	−0.11	−0.07	−0.09	0.03	−0.03	0.12	−0.01	0.08	−0.01	−0.04	−0.09	−0.04	0.06
Myrcene	−0.04	−0.05	−0.01	−0.05	−0.12	0.08	−0.14	−0.10	**−0.22**	−0.02	−0.05	0.01	0.01	−0.10	0.06	0.02	**−0.18**	−0.08	0.04
Thymol methyl ether	−0.04	0.07	−0.13	−0.01	0.00	0.05	−0.13	−0.03	−0.09	−0.08	0.02	0.03	0.06	0.04	−0.08	0.11	−0.09	−0.06	0.07
Carvacrol methyl ether	0.06	0.04	0.07	−0.12	0.09	0.03	0.02	0.06	0.05	0.06	0.00	−0.09	0.01	−0.01	−0.06	−0.04	−0.01	−0.04	0.09
β-Caryophyllene	0.13	0.05	0.09	0.05	**0.18**	0.01	**0.19**	**0.20**	**0.20**	0.08	0.16	−0.16	**0.17**	0.14	0.16	−0.11	**0.20**	0.16	0.10
β-Bisabolene	0.08	0.05	−0.05	0.00	0.15	0.00	0.12	0.15	0.12	0.04	**0.19**	−0.15	0.13	0.16	0.07	0.06	0.15	0.13	0.12
Caryophyllene oxide	**−0.22**	0.03	0.03	**−0.24**	0.00	−0.11	−0.04	−0.01	0.03	−0.01	−0.03	0.05	**−0.19**	**0.22**	**−0.33**	0.12	−0.01	−0.15	0.07

**Table 6:** Descriptive statistics of temperature (T), rainfall (R), sunshine duration (SS) and photosynthetically active solar radiation (PAR) in Lithuania climatic sub-districts in 2006–2016. Climatic sub-districts marking: Aukštaitija—A, Dzūkija—D, Mūša-Nevėžis—M-N, the Nemunas Lowland—NL, Venta—V, Sūduva—S, Žemaičiai—Z, Pajūris Lowland—PL, Pajūris—P. In Venta climatic sub-district temperature data are in 2012–2016; rainfall data—2008 and 2013–2016; in Aukštaitija climatic sub-district PAR data are in 2013–2016, in Mūša-Nevėžis and Venta climatic sub-districts PAR data are 2008–2016. The lines in the table means that data were not observed.

		Lithuania Climatic Sub-District								
		A	D	M-N	NL	V	S	Z	PL	P
T_june_, °C	Mean ± SD	16.3 ± 1.39	16.4 ± 1.26	16.1 ± 1.42	16.4 ± 1.31	15.1 ±1.67	16.3 ± 1.26	15.4 ± 1.41	15.9 ± 1.34	15.5 ± 1.34
	Min–max	14.4–18.4	14.5–18.1	14.3–18.4	14.8–18.4	13.8–17.5	14.5–17.8	13.6–17.5	14.1–17.6	13.3–17.5
	CV, %	9	8	9	8	11	8	9	8	9
T_july_, °C	Mean ± SD	18.8 ± 1.62	18.9 ± 1.36	18.9 ± 1.54	19.1 ± 1.47	17.8 ± 1.21	18.9 ± 1.36	18.2 ± 1.62	18.8 ± 1.52	18.8 ± 1.33
	Min–max	16.9–22.0	17.2–21.7	16.9–21.8	17.1–21.7	16.2–19.6	17.2–21.2	16.0–20.9	16.8–21.1	16.8–21.1
	CV, %	9	7	8	8	7	7	9	8	7
T_∑april–july_, °C	Mean ± SD	55.4 ± 2.86	55.8 ± 2.45	55.20 ± 2.68	56.60 ± 2.33	51.60 ± 3.24	56.10 ± 2.13	52.40 ± 2.51	54.60 ± 2.50	52.90 ± 2.02
	Min–max	50.2–59.9	51.3–60.0	49.7–58.7	51.7–59.4	46.5–54.0	51.5–58.7	46.4–54.8	48.8–57.9	48.2–55.8
	CV, %	5	4	5	4	6	4	5	5	4
R_june_, mm	Mean ± SD	64.4 ± 39.90	72.6 ± 40.27	64.4 ± 31.77	68.7 ± 28.87	52.9 ± 22.92	75.9 ± 29.26	63.4 ± 24.96	57.2 ± 22.36	49.2 ± 17.37
	Min–max	20.2–131.4	11.5–136.2	17.9–131.1	17.8–114.9	30.2–83.3	19.0–121.5	28.7–118.0	12.6–92.2	23.5–79.2
	CV, %	62	55	49	42	43	39	39	39	35
R_july_, mm	Mean ± SD	94.8 ± 28.37	102.1 ± 41.97	94.7 ± 33.50	92.00 ± 35.56	62.40 ± 31.50	114.80 ± 50.26	100.10 ± 49.10	99.23 ± 70.61	87.4 ± 50.5
	Min–max	52.4–135.0	24.8–165.0	35.3–141.0	44.3–166.0	30.1–165.0	26.8–192.1	21.6–180.7	16.9–286.0	11.0–193.7
	CV, %	30	41	36	39	50	44	49	71	58
R_∑Aptil–july_, mm	Mean ± SD	259.34 ± 59.38	283.00 ± 63.30	250.80 ± 63.51	249.00 ± 63.47	229.40 ± 72.78	292.10 ± 87.88	245.90 ± 64.15	234.59 ± 91.50	214.20 ± 66.76
	Min–max	166.2–386.8	207.1–388.1	134.8–338.8	149.9–324.8	121.3–275.5	151.2–464.5	125.8–345.9	102.0–454.9	89.3–333.0
	CV, %	23	22	25	25	32	30	26	39	32
SD_june_, h	Mean ± SD	264.9 ± 48.86	254.4 ± 52.99	266.8 ± 45.59	272.0 ± 55.18	–	282.1 ± 56.18	282.8 ± 42.19	287.1 ± 49.73	295.3 ± 26.26
	Min–max	180.1–319.3	168.3–321.3	184.8–322.1	192.4–364.2	–	181.8–364.2	207.3–341.7	217.6–353.4	258.2–334.9
	CV, %	18	21	17	20	–	20	15	23	9
SD_july_, h	Mean ± SD	264.1 ± 49.92	249.9 ± 50.02	272.1 ± 49.94	269.3 ± 52.71	–	273.4 ± 52.13	284.5 ± 49.52	282.5 ± 72.77	284.2 ± 52.37
	Min–max	177.4–343.5	176.8–342.3	199.3–361.3	186.5–395.5	–	199.9–341.7	222.90–373.10	171.6–396.0	192.6–356.1
	CV, %	19	20	18	20	–	19	17	26	18
SD_∑april–july_, h	Mean ± SD	1007.8 ± 102.93	945.8 ± 59.31	1012.4 ± 77.47	1022.6 ± 91.81	–	1056.6 ± 86.56	1064.0 ± 82.92	1080.2 ± 49.73	1096.3 ± 77.31
	Min–max	877.8–1246.5	848.7–1070.9	870.7–1149.9	895.8–1173.1	–	976.8–1264.2	959.8–1205.6	851.9–396.0	1008.7–122.8
	CV, %	10	6	8	9	–	8	8	5	7
PAR_june_, MJ/m^2^	Mean ± SD	334.7 ± 31.16	–	309.9 ± 43.78	323.7 ± 36.63	307.5 ± 31.94	–	–	331.5 ± 25.44	–
	Min–max	289.9 –358.3	–	217.7–346.5	261.1–369.8	264.0–339.4	–	–	291.5–371.8	–
	CV, %	9	–	14	11	10	–	–	8	–
PAR_july_, MJ/m^2^	Mean ± SD	299.3 ± 40.47	–	314.00 ± 22.59	312.80 ± 28.41	306.40 ± 32.93	–	–	319.30 ± 38.48	–
	Min–max	247.3–345.9	–	280.0–354.3	270.8–359.8	249.8–348.8	–	–	244.1– 369.7	–
	CV, %	14	–	7	9	11	–	–	12	–
PAR_∑april–july_, MJ/m^2^	Mean ± SD	1154.3 ± 32.07	–	1167.0 ± 27.05	1173.5 ± 33.84	1138.8 ± 61.00	–	–	1175.0 ± 105.32	–
	Min–max	1117.4–1182.5	–	1118.5–1202.7	1106.4–1218.2	1009.00–1214.0	–	–	909.7–1264.9	–
	CV, %	3	–	2	3	5	–	–	9	–

**Table 7:** Descriptive statistics of the main chemical compounds in *Thymus pulegioides* essential oils in different sub-districts of Lithuania. * denote statistically significant (*p* < 0.05) differences, ** denote statistically significant differences between habitats of Aukštaitija and the Nemunas Lowland climatic sub-districts by amount of nerol.

Lithuania Climatic Sub-District								
Chemical Compound		Aukštaitija	Dzūkija	Mūša-Nevėžis	Nemunas Lowland	Venta	Sudūva	Žemaičiai
Carvacrol	Mean ± SD, %	14.02 ± 6.01 *	19.78 ± 11.31	15.17 ± 7.98	19.32 ± 10.39	21.84 ± 14.52	27.73 ± 9.55 *	20.92 ± 7.94
	Min–max, %	4.79–29.30	5.30–48.00	0.00–32.26	0.00–40.36	11.02–43.95	17.37–39.95	10.94–43.27
	CV, %	43	57	53	54	66	34	38
Thymol	Mean± SD, %	4.74 ± 3.45 *	1.25 ± 1.69	3.70 ± 6.12	0.64 ± 1.39 *	0.37 ± 0.45	0.43 ± 0.67	4.52 ± 3.94
	Min–max, %	0.20–10.94	0.00–5.77	0.00–27.86	0.00–5.73	0.00–1.10	0.00–1.22	0.08–11.64
	CV, %	73	135	165	217	122	156	87
p-Cymene	Mean ± SD, %	17.12 ± 7.81 *	10.67 ± 6.97	12.12 ± 9.58 *	10.10 ± 8.18	0.99 ± 0.41 *	9.68 ± 1.23	11.59 ± 4.43
	Min–max, %	3.29–29.93	1.00–28.87	0.39–38.49	0.14–22.60	0.57–1.53	8.32–11.15	5.07–21.35
	CV, %	46	65	79	81	41	13	38
γ-Terpinene	Mean ± SD, %	12.97 ± 4.45 *	16.78 ± 9.46	15.53 ± 9.80 *	18.93 ± 7.74	10.75 ± 5.40 *	20.08 ± 2.96	23.92 ± 7.83 *
	Min–max, %	2.67–21.87	1.69–29.67	0.00–40.10	0.48–27.61	4.78–17.53	17.89–24.29	9.76–42.60
	CV, %	34	56	63	41	50	12	33
Carvacrol methyl ether	Mean ± SD, %	4.08 ± 1.65	3.33 ± 2.36	3.92 ± 2.29	3.47 ± 2.17	2.56 ± 2.07	4.19 ± 1.30	5.83 ± 2.02
	Min–max, %	1.00–6.04	0.00–8.56	0.12–9.26	0.00–7.99	0.00–5.70	2.60–5.73	1.18–9.17
	CV, %	40	71	58	63	81	31	35
Thymol methyl ether	Mean ± SD, %	1.63 ± 1.34	0.41 ± 0.52	0.66 ± 1.03	0.46 ± 0.53	0.18 ± 0.10	0.48 ± 0.60	1.21 ± 1.29
	Min–max, %	0.12–4.92	0.00–2.05	0.00–5.66	0.00–2.21	0.00–0.23	0.08–1.37	0.00–4.14
	CV, %	82	127	156	115	56	125	106
Gėraniol	Mean ± SD, %	6.93 ± 6.20	8.33 ± 10.10	8.43 ± 10.12	3.95 ± 5.57	15.91 ± 15.63 *	3.68 ± 6.68	1.48 ± 2.52 *
	Min–max, %	0.60–24.72	0.09–39.87	0.00–34.74	0.00–18.82	0.00–36.32	0.00–13.67	0.00–7.54
	CV, %	112	120	120	141	98	182	142
Geranialis	Mean ± SD, %	1.39 ± 1.26	1.68 ± 1.87	1.37 ± 1.63 *	0.52 ± 0.68	1.93 ± 1.88	0.51 ± 0.93	0.41 ± 0.65 *
	Min–max, %	0.10–4.71	0.00–5.03	0.00–6.57	0.00–2.15	0.00–4.28	0.00–1.89	0.00–1.88
	CV, %	91	111	119	131	97	182	159
Nerol	Mean ± SD, %	5.75 ± 5.27 *^,^**	5.73 ± 7.18 *	4.02 ± 4.66	1.50 ± 2.25 **	5.19 ± 5.24 *	1.28 ± 2.23	1.09 ± 1.63
	Min–max, %	0.51–17.17	0.00–20.57	0.00–16.71	0.00–8.78	0.00–12.40	0.00–4.78	0.00–5.39
	CV, %	92	125	116	150	101	182	150
Neral	Mean ± SD	4.35 ± 4.11	4.32 ± 5.53	3.97 ± 6.21 *	1.15 ± 1.74	4.10 ± 4.18	1.00 ± 1.89	0.75 ± 1.27 *
	Min–max	0.09–13.37	0.00–15.46	0.00–34.92	0.00–6.61	0.00–9.87	0.00–3.84	0.00–3.95
	CV, %	94	128	156	151	102	189	169
Linalool	Mean ± SD	0.99 ± 3.13	0.74 ± 1.37	2.45 ± 8.14	4.35 ± 12.93	0.41 ± 0.40	0.36 ± 0.10	0.29 ± 0.15
	Min–max	0.00–14.25	0.17–6.46	0.16–40.37	0.10–57.75	0.00–1.08	0.25–0.49	0.00–0.50
	CV, %	316	185	332	297	98	28	52
α-Terpinyl acetate	Mean ± SD, %	0.48 ± 0.45	0.56 ± 1.18	2.20 ± 6.84	2.90 ± 12.26	0.00	0.01 ± 0.02	0.004 ± 0.01
	Min–max, %	0.19–2.34	0.00–4.33	0.00–43.56	0.10–57.50	0.00	0.00–0.04	0.00–0.05
	CV, %	94	211	311	423	0.00	200	250
Myrcene	Mean ± SD, %	1.44 ± 0.42	1.70 ± 0.88	1.29 ± 0.52	1.38 ± 0.47	0.95 ± 0.48	1.41 ± 0.19	1.52 ± 0.35
	Min–max, %	0.93–2.74	0.62–4.19	0.00–2.33	0.54–2.44	0.39–2.68	1.14–1.60	0.73–1.96
	CV, %	29	52	40	34	51	13	23
β-Caryophyllene	Mean ± SD	4.99 ± 1.23	4.88 ± 1.00	5.47 ± 1.80	5.70 ± 2.10	6.02 ± 1.46	6.00 ± 1.39	6.48 ± 2.82
	Min–max	2.58–6.94	2.83–648	2.22–9.42	0.69–12.10	4.08–7.67	4.64–7.34	2.45–13.66
	CV, %	25	20	33	37	51	23	44
β-Bisabolene	Mean ± SD	3.05 ± 1.20	2.98 ± 0.96	2.73 ± 1.11 *	2.72 ± 0.80	2.97 ± 0.89	3.81 ± 1.26	3.76 ± 1.43 *
	Min–max	1.57–5.63	1.57–4.78	0.00–5.27	1.23–4.77	1.97–4.24	2.50–5.08	1.60–6.60
	CV, %	39	32	42	31	30	33	38
Caryophyllene oxide	Mean ± SD, %	1.73 ± 0.45 *	1.34 ± 1.15	1.19 ± 0.67 *	0.84 ± 0.39 *	2.97 ± 0.89	0.68 ± 0.20	0.79 ± 0.47 *
	Min–max, %	0.68–2.74	0.44–5.01	0.00–3.45	0.25–1.72	1.97–4.24	0.50–0.97	0.00–1.72
	CV, %	26	86	56	46	75	29	38
α-Terpinene	Mean ± SD, %	1.33 ± 0.52 *	1.50 ± 0.85 *	1.47 ± 0.81 *	1.89 ± 0.91	0.99 ± 0.41 *	1.76 ± 0.27	2.39 ± 0.95 *
	Min–max, %	0.23–2.59	0.00–2.48	0.00–2.95	0.04–3.86	0.57–1.56	1.60–2.16	1.16–4.85
	CV, %	39	57	55	48	41	29	40
Borneol	Mean ± SD	0.58 ± 0.33	0.66 ± 0.98	0.47 ± 0.34	0.39 ± 0.32	0.46 ± 0.29	0.64 ± 0.27	0.69 ± 0.53
	Min–max	0.00–1.20	0.00–4.65	0.00–2.95	0.04–1.21	0.00–0.79	0.46–1.04	0.00–2.19
	CV, %	57	148	72	82	63	42	77
Cis-β-Guaiene	Mean ± SD	1.14 ± 0.38 *	1.52 ± 0.61	1.54 ± 0.74 *	2.21 ± 1.43 *	1.88 ± 0.86	2.00 ± 0.57	1.78 ± 0.81
	Min–max	0.52–1.98	0.68–2.96	0.00–3.18	0.59–7.14	0.67–286	1.45–2.54	0.00–3.06
	CV, %	33	40	48	65	46	29	46

## Data Availability

Figure 1 was published in article Ložienė, K.; Vaičiulytė, V. Geraniol and carvacrol in essential oil bearing *Thymus pulegioides*: distribution in natural habitats and phytotoxin effect. *Molecules*. **2022**, *27(3)*: art no. 986.
